# Self-defensive nano-assemblies from camptothecin-based antitumor drugs

**DOI:** 10.1093/rb/rbv011

**Published:** 2015-08-10

**Authors:** Si-Yong Qin, Meng-Yun Peng, Lei Rong, Bin Li, Shi-Bo Wang, Si-Xue Cheng, Ren-Xi Zhuo, Xian-Zheng Zhang

**Affiliations:** ^1^Key Laboratory of Biomedical Polymers of Ministry of Education & Department of Chemistry, Wuhan University, Wuhan 430072, People’s Republic of China;; ^2^School of Chemistry and Materials Science, South-Central University for Nationalities, Wuhan 430074, People’s Republic of China

**Keywords:** self-assembly, camptothecin (CPT)-based drugs, morphology, hydrolysis

## Abstract

Camptothecin (CPT)-based drugs always undergo the reversible, pH-dependent lactone ring-opening reaction, yielding the inactive but toxic carboxylate form. Self-assembly strategy provides an effective route for preserving their bio-stability. In this article, nano-sized self-assemblies from CPT-based antitumor drugs were simply built up by directly diluting the stock dimethylsulfoxide solutions of (S)-(+)-CPT, (S)-10-hydroxyl camptothecin and carboxylic CPT with water/phosphate-buffered saline solution. Because of their different molecular structures in A-ring or modification on the 20-OH group, CPT self-assembled into helical nano-ribbons, whereas 10-hydroxycamptothecin and carboxylic CPT self-aggregated into flat nano-ribbons and cylindric nano-rods, respectively. Attractively, the self-assembly of CPT-based drugs could occur within 1 min at a low concentration of 1 × 10^−5 ^M. Adopting the J-type self-aggregation, self-assemblies were stable in aqueous solution and could effectively protect the CPT-based drugs from hydrolysis, which thereby kept their bioactivity for tumor therapy.

5th China-Europe Symposium on Biomaterials in Regenerative Medicine (CESB 2015) Hangzhou, China April 7–10, 2015

## Introduction

The emergence of abundant chemotherapeutic agents is undoubtedly one of the significant signs of progress in tumor treatments. Camptothecin (CPT), isolated from the *Camptotheca acuminate*, has been widely demonstrated to be a natural antitumor drug, which can inhibit the DNA topoisomerase I (topo I) enzyme and show remarkable antitumor activity against various tumor models [1–3]. With respect to the therapeutic mechanism, the A and E rings of CPT molecule may participate in the formation of CPT-topo I-DNA complex, which thereafter result in DNA double-stranded breaks [4]. In this regard, the structural features for CPT drugs are essential for their antitumor activity.

Because of the presence of the hydrolysable α-hydroxy-δ-lactone ring moiety, CPT-based drugs are vulnerable in aqueous media and the antitumor activity would rapidly decline once the drugs are administrated by oral or intravenous treatment. A representative example is topotecan, a most widely evaluated CPT derivative. The lactone ring of topotecan would undergo the rapid hydrolysis and only a small percentage of lactone rings remain intact after the administration for 30 min [2, 5]. The ring opening would lead to a reduced potency and yield an inactive and toxic carboxylate form [6, 7]. Additionally, the ring opening of lactone further results in the charged drug species, which afford poorer diffusibility through the cell lipid bilayer when compared with the close lactone form [8]. Therefore, the close lactone of CPT-based drugs is significant for the internalization of tumor cells and effective recognition to the topo I target.

To circumvent the drawbacks from hydrolysis, nanotechnology-assistant CPT drug delivery has been vastly exploited, which could effectively keep the CPT drugs intact and reduce the toxicity against normal tissues. Self-assembled micelles from amphiphilic polymers [9, 10] and polymer-prodrugs [11, 12] are reported to be a versatile nano-vehicle to deliver CPT-based drugs. Peptide amphiphilic nanofibers could also be introduced to encapsulate the hydrophobic CPT and to protect its biologically active lactone form for tumor therapy [13]. Very recently, CPT-containing low-molecular-weight pro-drugs have been proposed to stabilize the susceptible CPT drugs via a self-delivered manner [6, 14, 15]. Inspired by the studies that the π-conjugated property of CPT molecules could assist the self-assembly, we rationalize that the free CPT molecule and their derivatives have self-assembly abilities, which can protect the CPT-based antitumor drugs from the hydrolysis.

Keeping this in mind, free CPT and two derivatives were exploited to act as the self-assembled units to construct self-defensive nano-structures. Restricted by the structure–activity relationships that the C-D-E rings of CPT-based drugs cannot be altered [16], the ring backbone was untouched. 10-Hydroxycamptothecin (HCPT) with the modification on 10th position of the A-ring and the carboxylic camptothecin (CPT-COOH) obtained by esterification of the 20-OH group were proposed and their self-assembly was investigated. Because of the poor solubility of CPT-based drugs in aqueous solution, the strategy of dispersing the stock dimethylsulfoxide (DMSO) solutions into water/phosphate-buffered saline (PBS) was introduced and different self-assemblies from CPT and its derivatives were obtained. The drugs in these self-assemblies could be effectively shielded from the ring-opening hydrolysis, and their antitumor activities were therefore reserved.

## Experimental Section

### Materials

(S)-(+)-CPT and (S)-10-HCPT were purchased from Tianjin Heowes Biochem LLC. CPT-COOH was synthesized according to the literature [6]. DMSO was obtained from Shanghai Reagent Chemical Co. Bovine serum albumin (BSA) (concentration: 2.0 mg/ml in 0.9% aqueous NaCl solution containing sodium azide) was obtained from Invitrogen.

### Preparation of the self-assemblies

The stock DMSO solutions containing 10^−^^2^ M and 10^−^^3^ M CPT were prepared by directly dissolving a certain amount of CPT powder with DMSO under ultra-sonication. Then the stock solutions were stored in the dark at −20°C. Further dilutions were made by mixing aliquots of concentrated drug solution in DMSO with the corresponding amounts of PBS. Finally, the self-assemblies were obtained after shaking for 1 min at room temperature.

### Characterizations

Self-assembled morphologies from CPT-based drugs were investigated on a scanning electron microscope (SEM, Zeiss SIGMA FESEM). The freshly prepared solutions were equilibrated for 1 min for the rapid self-assembly, and SEM samples were obtained by dropping 1 μl of self-assemblies contained solution on a glass substrate. After slowly dried in air, the samples were coated with gold for the observation. Circular dichroism (CD) data were recorded on a J-810 spectropolarimeter (Jasco, Japan) with the DMSO or water solutions containing CPT-based drugs. The sample was prepared by diluting 1 × 10^−^^2^ M CPT stock DMSO solution to the final concentration of 1 × 10 ^− ^^5^ M for the CD measurement. Drugs (1 × 10^−^^5^ M) were poured into a 0.2 mm quartz cell and the data range was collected from 200 nm to 500 nm. The UV/Vis spectra in the wavelength range of 200–500 nm of the different self-assemblies were recorded on UV/Vis spectrophotometer (Perkin-Elmer Lambda Bio 40 UV/VIS spectrometer, USA). The samples were prepared by mixing the concentrated stock DMSO solutions with the corresponding amounts of PBS immediately before use. To ensure the same DMSO concentration in all preparations, additional amounts of DMSO were added, accordingly. Fluorescence spectra were collected with a LS55 luminescence spectrometry (Perkin-Elmer) with excitation wavelength at 365 nm and emission data ranging from 380 to 600 nm or with emission wavelength at 430 nm and excitation data ranging from 250 to 400 nm.

### Study on stability of CPT in the self-assemblies in PBS and BSA by reversed-phase high-pressure liquid chromatography

The lactone stability of CPT in the self-assembled helical nano-ribbons and nano-rods was investigated by analytical reversed-phase high-pressure liquid chromatography (HPLC) with a C18 column under ambient temperature. The eluting solvent was a linear gradient of CH_3_CN/H_2_O containing 0.05 M CH_3_COONH_4_. The timeline was set according to the literature [6] as follows: 0–5 min, 5–40% CH_3_CN; 5–20 min, 40–80% CH_3_CN; 20–23 min, 80–95% CH_3_CN and 23–25 min, 95–5% CH_3_CN. The existence of active lactone form and inactive carboxylate form was both detected at 360 nm with a UV-Vis detector. The reference HPLC traces of the active lactone form and inactive carboxylate form were determined in H_3_PO_4_–NaH_2_PO_4_ buffer (pH 3.0, 10 mM) and Tris-HCl buffer (pH 9.0, 10 mM). The lactone stability of CPT in the self-assembled nano-ribbons in PBS was measured by diluting the 10^−^^2^ M stock DMSO solution to 10^−^^4^ M in water and stood for 1 min, then further diluting to 10^−^^5^ M with PBS (pH 7.4, 10 mM) for 6 h and incubated at 37°C. The lactone stability of CPT in the self-assembled nano-rods was also investigated as control, which was obtained by diluting 10^−^^3^ M instead of 10^−^^2^ M stock DMSO solution to the desired concentration and incubated at 37°C for the same time. The discrepancy in the hydrolysis rate of lactone of CPT between two different self-assemblies was monitored by analytical HPLC. For the lactone stability of helical nano-ribbons in the BSA, 10^−^^2^ M stock DMSO solution was diluted to 10^−^^4^ M with water and stood for 1 min, then further diluting to 10^−^^5^ M with BSA for 3 h and incubated at 37°C; 50 µl of sample was withdrawn, diluted with 600 µl of cold methanol to precipitate the protein. After centrifuged at 5000 rpm for 5 min, 20 µl of the supernatant was then analyzed by HPLC. The lactone stability of nano-rods in BSA was also investigated as above.

## Results and Discussion

**Figure 1.**
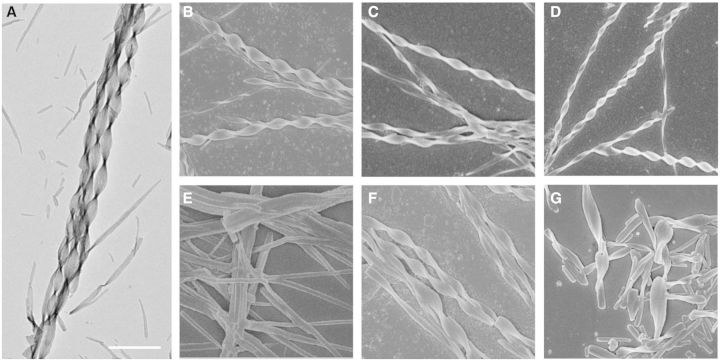
Transmission electron microscopy (**A**) and SEM images (**B–G**) of self-assembled helical nano-ribbons and their variants from natural CPT drug at different concentrations or with special treatments. Self-assembled helical nano-ribbons obtained by diluting the 10^−2^ M stock DMSO solution to 2.5 × 10^−4^ M (A, B), 5 × 10^−5^ M (C) and 1 × 10^−5^ M (D), respectively. Self-assembled nano-rods from CPT by diluting the 10^−3^ M stock DMSO solution to 1 × 10^−5^ M (E). The self-assembled nano-ribbons keeping their original morphology after standing for 2 days at 2.5 × 10^−4^ M aqueous solution (F). Treated by ultra-sonication for 2 min, the original long nano-ribbons were cut into short nano-ribbons (G). The scale bar is 1 µm.

The self-aggregation of natural CPT drug in solution was firstly judged based on the transformation from transparent solution to sub-transparent solution or hydrogel state, which was generally considered to be a visual evidence for the self-aggregation [17–19]. After mixing 1 × 10^−^^2^ M CPT stock DMSO solution with distilled water or PBS and making a concentration of CPT to 2.5 × 10^−^^4^ M, a sub-transparent solution was immediately formed, which suggested the rapid self-assembly of CPT molecules. Transmission electron microscopy and SEM images revealed that CPT molecules could self-assemble into well-defined nano-structures at a wide range of concentrations. As shown in Fig. 1A and B, the self-aggregation occurred within 1 min, and well-ordered helical nano-ribbons formed when the final concentration of CPT was 2.5 × 10^−^^4^ M. The average width and the length of the nano-ribbons were found to be around 300 nm and 10 μm, respectively. It should be noted that the helical nano-ribbons exhibited the right-handed helicity, which might be originated from the molecular chirality of CPT.

A low aggregation concentration of drug molecules for self-delivery is critically important because the exceeded drug concentration is extremely harmful for tumor treatments due to the unacceptable toxicity. Therefore, the self-assembled potential of CPT at low concentrations was further investigated by SEM. CPT exhibited robust self-assembled potential in aqueous solution, which was confirmed by the formation of nano-ribbons at the low concentrations of 5 × 10^−^^5^ M and even 1 × 10^−^^5^ M, respectively. Similar nanoribbon bundles were also observed by SEM images in Fig. 1C and D. However, at the concentrations of the 5 × 10^−^^5^ M and 1 × 10^−^^5^ M, the average widths of self-assembled nanoribbons were smaller than that formed at 2.5 × 10^−^^4^ M. The plausible reason was attributed to the concentration-dependent sizes for the nano-ribbons. Furthermore, the self-assembled morphology can be tunable by the original CPT concentration in the stock DMSO solution. The cylindric nano-rods instead of helical nano-ribbons were formed when diluting 1 × 10^−^^3^ M CPT stock DMSO solution to the same final concentration of 1 × 10^−^^5^ M (Fig. 1E). The different self-assembled morphologies indicated that the instantaneous concentration for interacting with the solvent was critical for the formation of helical nano-ribbons. To verify the inference, ultrasonic dispersion was introduced to rapidly disperse the stack DMSO solution into water. Although the stock solution with 1 × 10^−^^2^ M CPT was used to prepare the samples, CPT molecules could hardly self-assemble into the nano-ribbons but self-aggregated into some fiber-like nano-structures. These findings indicated that a certain instantaneous concentration for the dispersion was required for the helical structure.

To estimate the stability of the self-assembled helical nano-ribbons in the aqueous solution, the corresponding change of the macroscopical solution and microscopic morphologies of the self-assemblies were monitored. After 2 days, the prepared sub-transparent solution containing the self-assemblies could still keep the colloidal solution state, which was further investigated, and helical nano-ribbons were also found under SEM (Fig. 1F), indicating that the nano-ribbons were stable and could keep their original morphology without disruption for several days at room temperature. It was reported that the carboxylate form of CPT molecules hydrolyzed from the active lactone form was water soluble and therefore could not self-assemble into any aggregates [20]. Therefore, the emergence of the nano-ribbons suggested that the CPT drug still kept its intact lactone form. The self-assembled nano-ribbons could not only effectively protect the CPT drug from hydrolysis, which was critical for keeping the bioactivity, but also might exhibit enhanced cellular uptake levels relative to the single molecular state of CPT due to enhanced permeability and retention effect. To further investigate the stability of the nano-ribbons, the tolerance of nano-structure to external disturbance was conducted employing ultra-sonication as the stimulus. Although the self-assemblies were almost cut into short segments, the short nano-ribbons were also observed when treated with the ultra-sonication for 2 min (Fig. 1G). Compared with the long helical nano-ribbons with micro-scale lengths, the short ones may achieve more effective cell endocytosis and therefore exhibit effective tumor therapy for both *in vitro* and *in vivo* applications.

**Figure 2.**
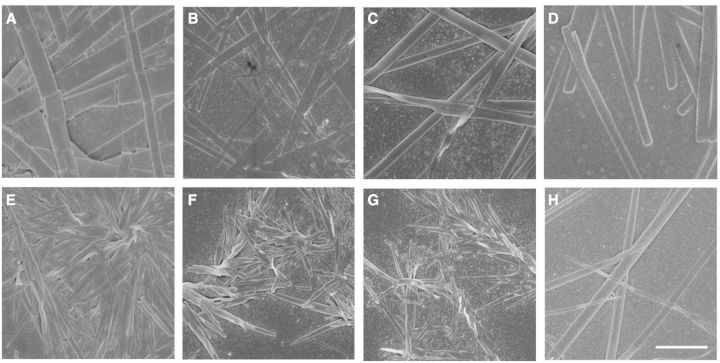
Self-assembled nano-structures from HCPT (**A–D**) and CPT-COOH (**E–H**) at different concentrations or with different sample preparing methods. Self-assembled flat nano-ribbons from HCPT at the concentrations of 2.5 × 10^−4^ M (A), 5 × 10^−5^ M (B); nano-rods (C, D) at 1 × 10^−5^ M; short and dense nano-rods from CPT-COOH at concentrations of 2.5 × 10^−4^ M (E), 5 × 10^−5^ M (F) and 1 × 10^−5^ M (G) and long and sparse nano-rods at 1 × 10^−5^ M. (A–C) and (E-G) the samples prepared by diluting the 10^−2^ M stock DMSO solution of drugs to the pre-setted concentrations. (D) and (H) the samples prepared by diluting the 10^−3^ M stock DMSO solution to 1 × 10^−5^ M. The scale bar is 1 µm.

Beside CPT, the self-assembled ability of HCPT and CPT-COOH was also investigated, accordingly. As shown in Fig. 2A and B, HCPT self-assembled into flat nano-ribbons at the concentrations of 2.5 × 10^−^^4^ M, 5 × 10^−^^5^ M, which were slightly different from the nano-rods self-assembled from 1 × 10^−^^5^ M CPT solution (Fig. 2C and D). Similar to the helical nanoribbons from CPT drug under different concentrations, the width of flat nano-ribbons also exhibited concentration-dependent. Nevertheless, by both diluting 1 × 10^−^^2^ M and 1 × 10^−^^3^ M stock solutions to 1 × 10^−^^5^ M, HCPT self-assembled into the nano-rod-like nanostructures. The different nanostructures from CPT by diluting different stock DMSO solutions to the same final concentration were not observed for HCPT, which might be ascribed to the different self-assembled abilities. HCPT exhibited more robust self-assembled potential than CPT [4], and the difference in the original concentrations of 1 × 10^−^^2^ M and 1 × 10^−^^3^ M could not lead to the transformation in the self-assemblies.

By diluting the 10^−^^2^ M CPT-COOH stock DMSO solution to the corresponding concentrations, CPT-COOH self-assembled into short and dense nano-rods at all observed concentrations of 2.5 × 10^−^^4^ M, 5 × 10^−^^5^ M and 1 × 10^−^^5^ M (Fig. 2E–G). However, diluting the 10^−^^3^ M CPT-COOH stock DMSO solution to 1 × 10^−^^5^ M, CPT-COOH self-assembled into long and sparse nano-rods (Fig. 2H). This result was attributed to the fact that the CPT-COOH molecules would rapidly self-aggregate at high instantaneous concentrations, yielding the thick nano-rods with short lengths. Also the CPT-COOH molecules in diluted solution could engage in the long-range order arrangement because of the weak interaction within CPT-COOH molecules.

In consideration of the potential application of the CPT-based drugs in tumor therapy, the self-assembled behavior of CPT-based drugs was also investigated in PBS (pH 7.4, 10 mM), which was used to mimic the physiological environment in the body. CPT and HCPT could also self-assemble into helical nano-ribbons and flat nano-ribbons, respectively. There was no apparent difference in the morphologies for CPT and HCPT in both water and PBS. Nevertheless, CPT-COOH could only self-aggregate into some random structures, which was mainly attributed to that the salts in the PBS had a significant influence on the carboxyl group of CPT-COOH. The electrostatic repulsion from ionized carboxyl groups inhibited the well-ordered self-assembly.

**Figure 3.**
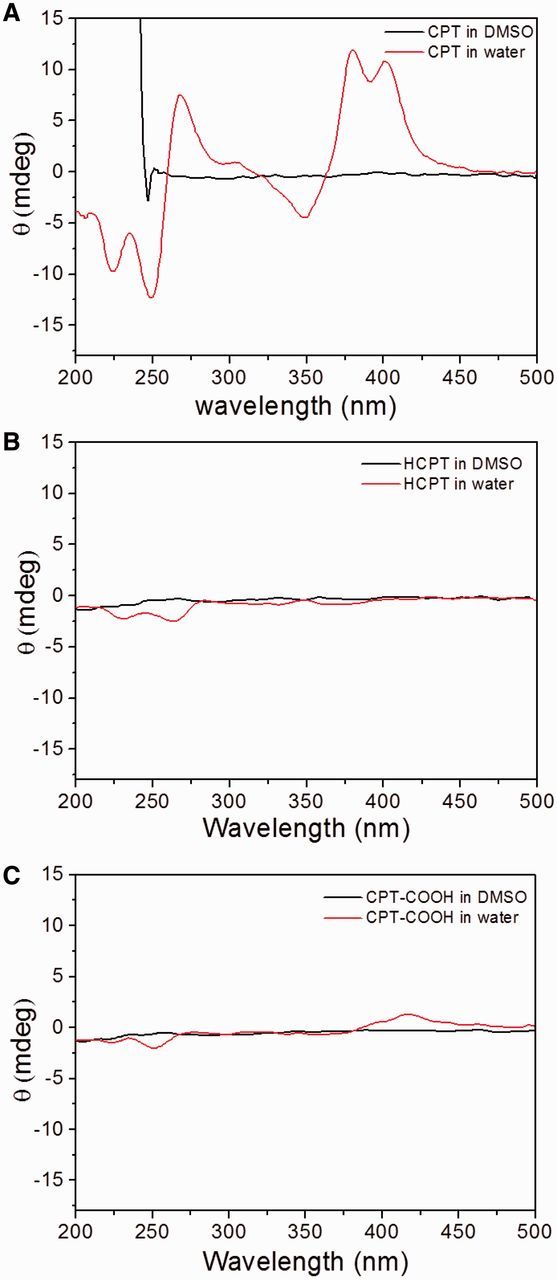
The CD spectra of CPT-based drugs in DMSO and water at the same final concentration of 1 × 10^−5^ M.

To investigate the special right-handed helical structure in the nano-ribbons, CD was exploited to measure the characteristic absorption. It was found that the CD spectra of CPT drugs displayed different signals in aqueous and DMSO solutions. As shown in Fig. 3, self-assembled CPT nano-ribbons displayed some strong absorption regions between 200 nm and 500 nm, and the obvious Cotton effects could be observed due to the presence of couplet signals centered at 259 nm and 362 nm. The bisignate CD signals were indicative of the strong chiral arrangement in our nano-ribbons [15, 21]. Also, the essential feature of positive chirality further verified the right-handed helical orientation of CPT molecules. Beside a strong random peak at the low wavelength, however, no characteristic CPT absorption signals were observed for the CPT drug in DMSO, where the drug molecules were expected to exist in a monomeric form. In addition, no strong CPT absorption signals were found in the CD spectra of HCPT and CPH-COOH drugs (Fig. 3B and C), indicating the absence of helical structures in these self-assemblies from HCPT and CPH-COOH. The result was in accordance with the SEM observation that the right-handed helix was only found in the nano-ribbons from natural CPT drug. Those findings suggested the chirality of chiral nanostructures was not only determined by the chirality of chiral molecules but also limited by external environment and the steric hindrance.

**Figure 4.**
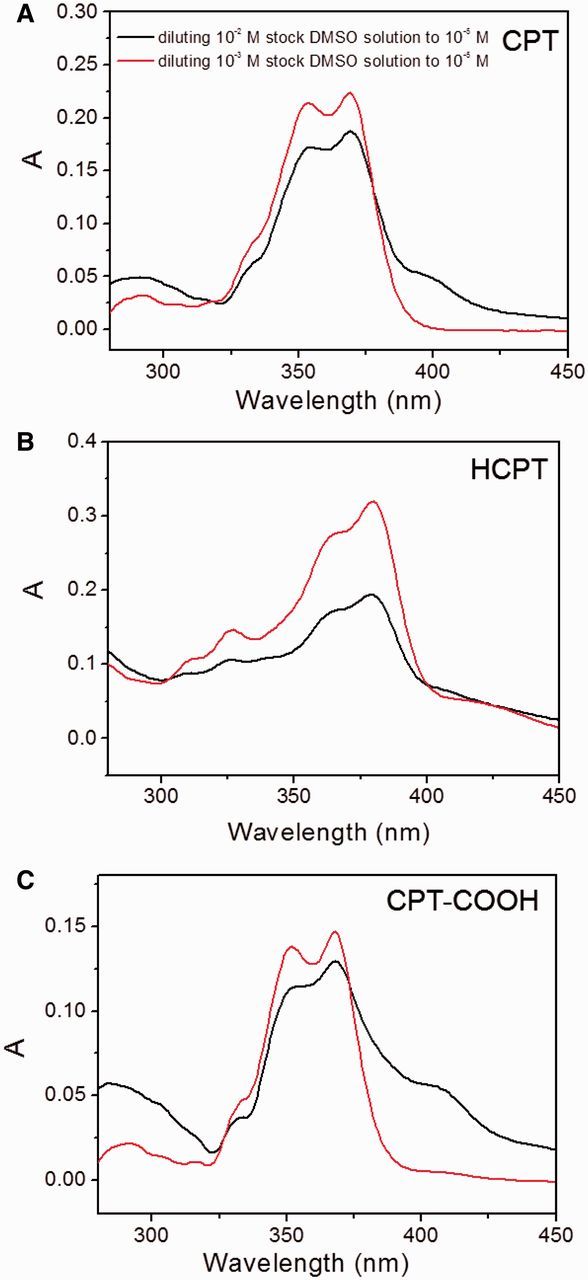
The UV-Vis spectra of CPT-based drugs at the same final concentration of 1 × 10^−5^ M prepared by diluting the 1 × 10^−2^ M (the bottom line) and 1 × 10^–3^ M (the top line) stock solutions of CPT, respectively.

The understanding of molecular arrangement in the self-assemblies is in favor of protecting the bioactivity of the drug and designing the drug delivery systems. To excavate the self-assembled mechanism for the CPT-based drugs, the self-aggregation fashion in the CPT drugs was investigated. It was generally reasonable that the same CPT concentration would display the identical UV-vis and fluorescent spectra. A comparison of the UV-vis absorption of two CPT aqueous solutions with the same concentration of 1 × 10^−^^5^ M by diluting either 1 × 10^−^^2^ M or 1 × 10^−^^3^ M stock DMSO solutions was firstly conducted. Attractively, the UV-vis spectra displayed remarkable differences in the extinction coefficients and the numbers of the central peaks. Compared with the UV-vis spectrum from the sample prepared from 1 × 10^−^^2^ M stock solution, the sample prepared from 1 × 10^−^^3^ M stock had stronger absorption at centered peaks of 353 nm and 369 nm (Fig. 4A). Moreover, a new absorption peak around 400 nm was detected from the solution prepared from the more concentrated stock solution. The different extinction coefficients from two stock solutions could also be detected in HCPT and CPT-COOH drugs (Fig. 4B and C). However, diluting the 1 × 10^−^^2^ M stock solution to 1 × 10^−^^5^ M did not resulted in new absorption peak as shown in the UV-vis spectra of HCPT (Fig. 4B). The finding was in agreement with the previous SEM images, indicating that HCPT self-assembled into similar nano-rods even though from different original concentrations. It may further indicate that HCPT has more robust self-assembled potential than CPT. The abnormal UV-vis absorption for CPT drugs in aqueous solution suggested that CPT molecules and their analogs may exist in the aggregated states but not the monomeric forms. The results supported the SEM observation that CPT-based drugs could self-assemble into nano-structures in spite of the different stock concentrations. Nevertheless, the different UV-vis absorption spectra of CPT-based drugs indicated the drug molecules from the concentrated stock solution may have stronger self-assembled ability, which was evidenced by the appearance of the new absorption peak around 400 nm.

**Figure 5.**
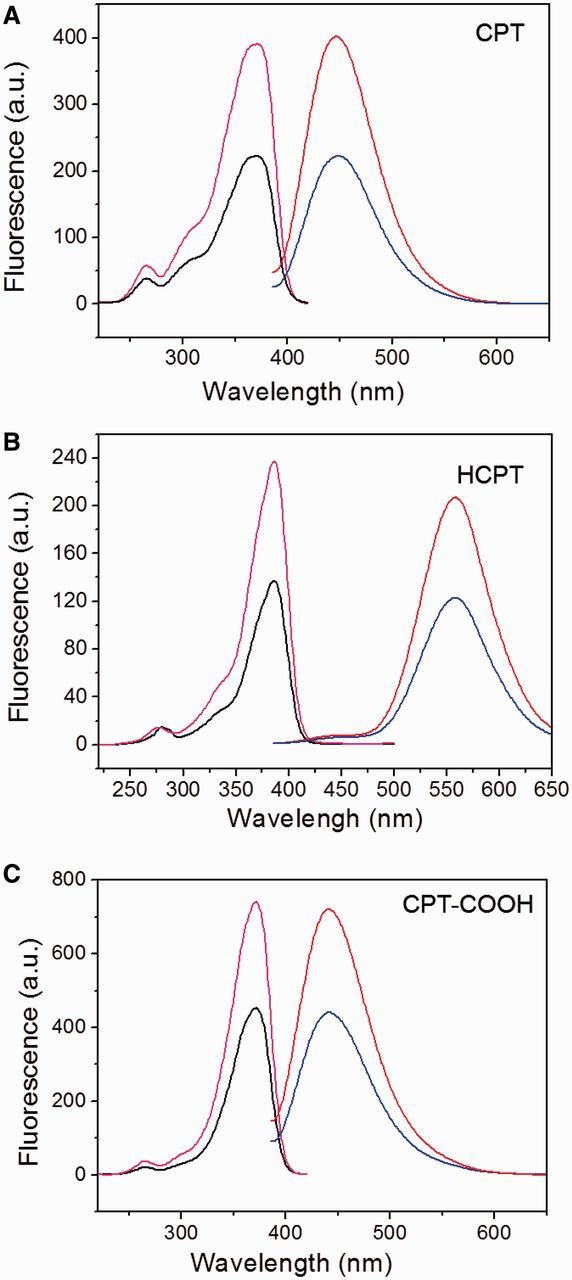
The fluorescence excitation (left lines) and emission (right lines) spectra recorded from the samples prepared by diluting the 1 × 10^−2^ M (the bottom lines) and 1 × 10^−3^ M (the top lines) stock solutions of CPT to 1 × 10^−5^ M in water. The fluorescence detector excitation and emission settings: *λ*_ex_ = 370 nm and *λ*_em_ = 435 nm, respectively.

Fluorescent spectra of the samples prepared by two strategies were also collected. The fluorescent emission and excitation spectra of self-assembled helical nano-ribbons were also found to be near 50% less intense than that the self-assembled cylindric nano-rods obtained by diluting 1 × 10^−^^3^ M stock solution (Fig. 5A). The fluorescent differences of CPT drug from two different prepared methods were also observed for HCPT and CPT-COOH (Fig. 5B and C). The finding, together with UV-vis absorption in Fig. 4, indicated the J-type self-aggregation in the all observed drugs [4].

**Figure 6.**
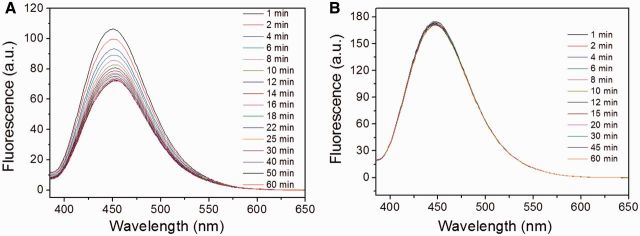
The kinetic study of the self-assembled helical nano-ribbons (**A**) and cylindric nano-rods (**B**) by the fluorescence emission spectra recorded from the samples prepared by diluting the 1 × 10^−2^ M and 1 × 10^−3^ M stock solutions of CPT to 1 × 10^−5^ M in water, respectively.

As mentioned above, the fluorescent intensity would diminish upon aggregation of molecules, which may provide a facile strategy to investigate the self-assembled kinetics. The self-assembled helical nano-ribbons and cylindric nano-rods from diluting the 1 × 10^−^^2^ M or 1 × 10^−^^3^ M stock solutions of CPT down to 1 × 10^−^^5^ M in water were selectively investigated by fluorescence spectra. As shown in Fig. 6, the fluorescent intensity exhibited time-dependent decrease within the first 30 min and intended to remain constant as increasing the time. This was possibly attributed to the rapid self-assembly for the CPT molecules and the self-aggregation completed within 30 min. As for the self-assembled cylindric nano-rods, the fluorescence intensity kept almost constant within the observed time. The possible reason was that self-assembly occurred too quickly to monitor the fluorescent change. There was another possibility that the self-assembled potential was relatively weak, and the J-type self-aggregation could not lead to the fluorescent change. The rapid self-assembly correlated fairly well with the observation that the self-assemblies could be immediately formed once injecting DMSO solution of CPT into water/PBS. The rapid self-assembly is critical important for the hydrolysable drugs, which could quickly protect them from deactivation.

**Figure 7.**
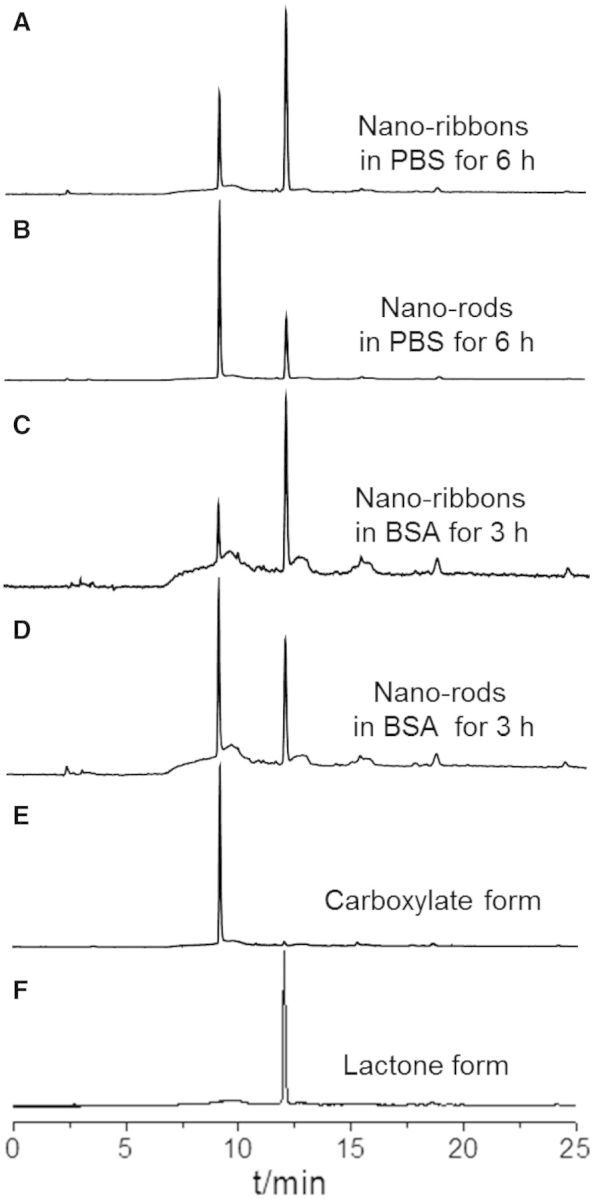
HPLC traces of CPT molecules in the helical nano-ribbons and cylindric nano-rods after incubation with PBS for 6 h (**A**, **B**) or BSA for 3 h (**C**, **D**). The CPT molecules existed in the carboxylate form at pH 9 (**E**) and the lactone form at pH 3 (**F**) were recorded as control.

**Scheme 1.**
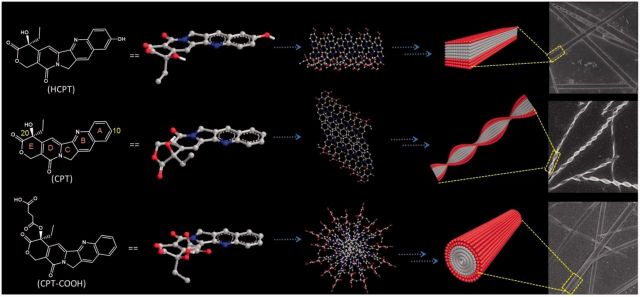
Proposed self-assembly mechanisms of CPT-based drugs. Chemical structures of CPT drugs and their ball and stick model in ChemBio3D, as well as the proposed arrangement of the units in the nano-sized self-assemblies were illustrated. Driven by the π-π interaction from the neighboring CPT quinoline rings of the adjacent conjugates, HCPT adopted the side-to-side arrangement to self-assemble into the flat nano-ribbons; CPT molecules self-assembled into helical nano-ribbons via an edge-to-edge pattern; The hydrophobic quinoline rings within the CPT-COOH molecules rapidly aggregated away from water and the cylindric nano-rods were formed for CPT-COOH.

Based on the above spectra analysis, probable self-assembled mechanisms for the CPT-based drugs were proposed. Driven by the π-π interaction from the neighboring CPT quinoline rings of the adjacent conjugates, CPT molecules begun to aggregate via an edge-to-edge pattern (J-type self-aggregation) and self-assembled into the helical nano-ribbons (Scheme 1). The hydrogen bond may also participate in the formation of the observed nano-structures [20], although the exact mechanism was not very clear and required further evaluation. Although the J-type self-aggregation was also found in HCPT-based self-assemblies, the introduction of hydrophilic carboxyl group to the 10-position of CPT, which might in some extent prevent the edge-to-edge helical arrangement but adopted the side-to-side arrangement to self-assemble into the flat nano-ribbons. The incorporation of succinic anhydride to CPT molecule resulted in the steric hindrance, which also prevented the well-ordered arrangement. The hydrophobic quinoline rings within the CPT-COOH molecules rapidly aggregated away from water to minimize the total energy, leaving the hydrophilic carboxyl groups exposed to the aqueous environment. Therefore, the cylindric nano-rods were formed by diluting the concentrated CPT-COOH stock solution with water.

It is important to note that CPT-based drugs always undergo the reversible, pH-dependent lactone ring-opening reaction, yielding the inactive but toxic carboxylate form. The lactone hydrolysis is especially enhanced with the co-existence of proteins, which could bind and sequester the carboxylate form [22]. Self-assembly strategy provides an effective route for keeping their bioactivity. Therefore, the stability of the self-assemblies was investigated by HPLC at 37°C. It was difficult to obtain the monomeric form of CPT in the concentration range for the HPLC quantitative determination. Thus, two different self-assemblies instead of the monomeric form and its corresponding aggregated state were measured. In contrast to the self-assembled nano-rods from diluting the 1 × 10^−^^3^ M CPT stock solution, 72.1% of which underwent hydrolysis to the inactive carboxylate form, helical nano-ribbons from diluting the 1 × 10^−^^2^ M CPT stock solution was 59.9% intact when stored at 1 × 10^−^^5^ M in PBS for 6 h Fig. 7. Meanwhile, after the binding and sequestration by BSA for 3 h, 60.9% of self-assembled nano-rods hydrolyzed into the inactive carboxylate form, while only 37.6% of CPT in the helical nano-ribbons underwent the ring-opening reaction. Those findings suggested the nano-ribbons could more effectively protect the CPT drugs than the nano-rods, which also coincided with the results from the fluorescent and UV-vis spectra, further indicating the helical nano-ribbons were more stable than the self-assembled nano-rods.

## Conclusions

In summary, the CPT-based drugs exhibited strong self-assembly potential in a wide range of concentrations and different nano-sized self-assemblies from CPT-based drugs were simply and rapidly built up by injecting the stock DMSO solutions into water/PBS. Self-assembly of CPT, HCPT and CPT-COOH resulted in helical nano-ribbons, flat nano-ribbons and short nano-rods, respectively, at a concentration of 5 × 10^−^^5^ M. The helical nano-ribbons could keep their original morphology for several days and exhibited ultrasonication tolerance, which suggested that the self-assemblies were stable in aqueous solution. HPLC result further indicated that the self-assemblies could effectively protect the CPT-based drugs from hydrolytic lactone opening, which thereby kept their bioactivity for tumor therapy. Fluorescent spectra data indicated the J-type self-aggregation was the main arrangement pattern for all the self-assemblies. Meanwhile, ultra-sonication technique may be used to adjust the lengths of the self-assemblies for potential applications. The investigation on drug self-assembly could enable us further understand the mechanism of self-assembly, which gives insight into designing the self-delivered drug delivery systems.

## References

[1] Hanson BA Schowen RL Stella VJ A mechanistic and kinetic study of the E-Ring hydrolysis and lactonization of a novel phosphoryloxymethyl prodrug of camptothecin. Pharm Res 2003;20:1031-8. 12880289 10.1023/a:1024410322870

[2] Yount G Yang Y Wong B A novel camptothecin analog with enhanced antitumor activity. Anticancer Res 2007;27:3173-8. 17970058

[3] Cui L Ni X Ji Q Co-overexpression of geraniol-10-hydroxylase and strictosidine synthase improves anti-cancer drug camptothecin accumulation in *Ophiorrhiza pumila*. Sci Rep 2015;5:8227. 25648209 10.1038/srep08227 PMC4316170

[4] Nabiev I Fleury F Kudelina I Spectroscopic and biochemical characterisation of self-aggregates formed by antitumor drugs of the camptothecin family. Biochem Pharmacol 1998; 55:1163-74. 9719470 10.1016/s0006-2952(97)00508-x

[5] Fassberg J Stella VJ A kinetic and mechanistic study of the hydrolysis of camptothecin and some analogues. J Pharm Sci 1992;81:676-84. 1403703 10.1002/jps.2600810718

[6] Kim SH Kaplan JA Sun Y The self-assembly of anticancer camptothecin-dipeptide nanotubes: a minimalistic and high drug loading approach to increased efficacy. Chem Eur J 2015;21:101-5. 25384556 10.1002/chem.201404520

[7] Yin Q Tong R Yin L Anticancer camptothecin-N-poly(lactic acid) nanoconjugates with facile hydrolysable linker. Polym Chem 2014;5:1581-5. 26005498 10.1039/C3PY01245J PMC4439007

[8] Hatefi A Amsden B Camptothecin delivery methods. Pharm Res 2002;19:1389-99. 12425455 10.1023/a:1020427227285

[9] Bian F Jia L Yu W Self-assembled micelles of N-phthaloylchitosan -g-polyvinylpyrrolidone for drug delivery. Carbohydr Polym 2009;76:454-9.

[10] Tang DL Song F Chen C A pH-responsive chitosan-b-poly(p-dioxanone) nanocarrier: formation and efficient antitumor drug delivery. Nanotechnology 2013;24:145101. 23481178 10.1088/0957-4484/24/14/145101

[11] Wang H Tang L Tu C Redox-responsive, core-cross-linked micelles capable of on-demand, concurrent drug release and structure disassembly. Biomacromolecules 2013;14:3706-12. 24003893 10.1021/bm401086d PMC4232441

[12] Zhou Z Ma X Jin E Linear-dendritic drug conjugates forming long-circulating nanorods for cancer-drug delivery. Biomaterials 2013;34:5722-35. 23639529 10.1016/j.biomaterials.2013.04.012

[13] Soukasene S Toft DJ Moyer TJ Antitumor activity of peptide amphiphile nanofiber-encapsulated camptothecin. ACS Nano 2011;5:9113-21. 22044255 10.1021/nn203343z PMC3229267

[14] Lin YA Cheetham AG Zhang P Multiwalled nanotubes formed by catanionic mixtures of drug amphiphiles. ACS Nano 2014;8:12690-700. 25415538 10.1021/nn505688b PMC4334259

[15] Cheetham AG Zhang P Lin Y Supramolecular nanostructures formed by anticancer drug assembly. J Am Chem Soc 2013;135:2907-10. 23379791 10.1021/ja3115983 PMC3609949

[16] Verma RP Hansch C Camptothecins: a SAR/QSAR study. Chem Rev 2009;109:213-35. 19099450 10.1021/cr0780210

[17] Qin SY Jiang HF Peng MY Adjustable nanofibers self-assembled from an irregular conformational peptide amphiphile. Polym Chem 2015;6:519-24.

[18] Qin SY Chu YF Tao L Controllable micro/nanostructures via hierarchical self-assembly of cyclopeptides. Soft Matter 2011;7:8635-41.

[19] Li J Gao Y Kuang Y Dephosphorylation of _D_-peptide derivatives to form biofunctional, supramolecular nanofibers/hydrogels and their potential applications for intracellular imaging and intratumoral chemotherapy. J Am Chem Soc 2013;135:9907-14. 23742714 10.1021/ja404215g PMC3730259

[20] Ma M Xing P Xu S Reversible pH-responsive helical nanoribbons formed using camptothecin. RSC Adv 2014;4:42372-5.

[21] Hembury GA Borovkov VV Inoue Y Chirality-sensing supramolecular systems. Chem Rev 2008;108:1-73. 18095713 10.1021/cr050005k

[22] Opanasopita P Yokoyamab M Watanabe M Influence of serum and albumins from different species on stability of camptothecin-loaded micelles. J Control Release 2005;104:313-21. 15907582 10.1016/j.jconrel.2005.02.014

